# Prevalence of Musculoskeletal Disorders among Iranian Steel Workers

**Published:** 2012-04-01

**Authors:** M Aghilinejad, A R Choobineh, Z Sadeghi, M K Nouri, A Bahrami Ahmadi

**Affiliations:** 1 Occupational Medicine Research Center (OMRC), Tehran University of Medical Sciences, Tehran, Iran; 2 Department of Occupational Health, Research Center for Health Sciences, School of Health and Nutrition, Shiraz University of Medical Sciences, Shiraz, Iran

**Keywords:** Musculoskeletal disorder, Prevalence, Steel worker, Nordic questionnaire, Ergonomic program

## Abstract

**Background:**

Musculoskeletal disorders in developing countries are considered as main cause of occupational disorders and disability and highly associated with socioeconomic burden to individual, organization and society in general view. The purpose of this study was to determine the prevalence of musculoskeletal disorders and associated risk factors among Iranian steel workers.

**Methods:**

In a cross-sectional study, 1439 questionnaires were provided from 1984 randomly selected workers of four Iranian steel industries. Data of musculoskeletal disorders was gathered by means of standardized Nordic self-reporting questionnaire. Demographic and work related data were collected into the check list.

**Results:**

Out of 1984 individuals, 1439 questionnaires returned and mean age of study workers was 37.23±8.74 years old. Among workers, 46.3% in the past week and 61% in the last year claimed one of musculoskeletal disorders in their bodies. Lumbar, knee(s) and neck areas had the most common musculoskeletal disorders. Musculoskeletal disorders had significant association with the job time of work and BMI.

**Conclusion:**

Musculoskeletal disorders in Iranian steel industries happened in high rate. Ergonomic interventions strategies into the workplaces must be focused to eliminate environmental hazards such as apposition on the time of work and manual handling of heavy loads.

## Introduction

Musculoskeletal disorders were presented as increasing burden in several societies. Health policy makers and other professionals search for finding suitable national preventive programs for musculoskeletal disorders reporting and prevention;[1][2] Repetitive tasks and awkward position are known as work related factors and age, gender and psychological characters are known as worker related risk factors of musculoskeletal disorders among workers.[3][4][5][6][7]

In developing countries, workplace related disorders caused several problems.[3] In the general view, musculoskeletal disorders have great impacts on industries and society more than workers themselves. Some previous studies assessed the prevalence of musculoskeletal disorders among Iranian workers in some industries;[8][9][10][11] and as example, we can note to Alipour et al. study on more than 14,000 workers of Iranian automobile factory.[8]

Steel factories are basic industries in each country and this importance is increasing in developing countries such as Iran. Workers in these companies are directly involved in production process and physical activities such as manual material handling and awkward postures are very common.

The prevalence of musculoskeletal disorders among workers followed two main purposes: Detecting musculoskeletal disorders’ prevalence rate and finding causative and other relative factors which had impact on this rate. The present study was performed for evaluation of the musculoskeletal disorders prevalence associated risk factors among Iranian steel workers.

## Materials and Methods

In this cross sectional study, the inclusion criteria were only full-time working in four main metal industries (n=14373) with at least one year job experience. We considered past medical history of workers and excluded workers with previous non-work related musculoskeletal disorders in their health folders or any health conditions or disorders which might have impact on musculoskeletal system except their work. We selected study workers (n=1984) among them with multi stages randomized sampling method and all workers had same work duties.

Workers of each steel industry according to number of its workers had chance for participation in the study and we distributed 1984 questionnaires and finally 1439 (response rate: 72.53%) questionnaires returned. Incomplete questionnaires and failure in their returning were main causes of noted response rate. Musculoskeletal disorders related complaints were defined as pain or discomfort experienced in the different body regions that continued for at least a few hours during the past week or year.

After approving study in Ethical Committee of Occupational Medicine Research Center of Tehran University of Medical Sciences and Health Services, a cover sheet was attached to the front of Nordic musculoskeletal questionnaire (NMQ) and we demonstrated our study and instruction for completion of forms. Our forms were distributed and collected during one week. We had no penalties or rewards for participations in the study and researchers were ready to answer to all of their questions. Informed consent was implied when questionnaires were voluntarily completed and returned.

Data were gathered by means of standardized Nordic self-reporting questionnaire.[12] The NMQ was developed from a project funded by the Nordic Council of Ministers and included questions such as age, job duration, weight of carried loads, daily working hours and musculoskeletal complaints in each of the following body regions: neck, shoulder, elbow, wrist/hand, upper back, lumbar, one or both hips/thighs, one or both knees and one or both ankle/feet. Data on daily working hours were obtained by the time spent in the workplace. The validity and reliability of the questionnaire were investigated and approved in different studies and several languages, including the Persian language.[10][13] This questionnaire was used as questionnaire or interview device.[14] The NMQ was used in several studies for evaluating musculoskeletal problems, including computer and call centre workers;[15] car drivers;[16] coopers in the whisky industry;[17] and forestry workers.[18] However, medical examination is essential to establish a clinical diagnosis.[19][20]

Musculoskeletal complaint was defined as pain or discomfort experienced in soft tissue of the different body regions, which had occurred at least 2-3 work days during the past week or the last 12 months. All medical examination and questionnaire filling were supervised by the research team.

Chi Square test was used to compare demographic variables between workers with and without musculoskeletal disorders. A multinomial regression model was used to clarify the differences. In this model, one of musculoskeletal disorders in recent week and year was selected as a dependent variable. Demographic variables including age, sex, dominant hand, past job history and BMI were inserted in the model. A backward (Likelihood ratio) procedure was used in this analysis too. Calculations were done using the SPSS software (SPSS Inc., version 16, Chicago IL, USA) and p-value less than 0.050 was considered significant.

## Results

The average age was 37.23±8.74 years and 1398 (97.2%) workers were male. Subjects worked in the company for an average of 56 hours (at least one working shift) per week and he average of their career duration was 13.5±8 years (range: 1-40 years). Among our workers, 1317 (91.5%) were right-handed and mean of their BMI was 25.67±3.55. Details of demographic variables were presented in Table 1.

**Table 1:** Some of demographic details of our include subjects (n=1439).

**Personal characters**	**Mean/frequency**	**Standard deviation/percentage**	**Min-Max**
Age (year)	37.23	8.74	20-64
Body mass index (Kg/m^2^)	25.68	3.56	16.41-58.59
Career duration (year)	13.5	8.09	1-40
Sex (male)	1398	97.2	-

According to results of Nordic musculoskeletal disorders questionnaire, one week and 12-month period-prevalence of musculoskeletal disorders at any of the four body sites of included workers were 46.3% and 61% respectively. Musculoskeletal disorders in last week were most commonly reported at the lumbar (63.81%), followed by the knee(s) (45.35%), neck (39.79%), back (30.03%) and in 12-months period, these rates were most commonly reported at the lumbar (64.12%), followed by the knee(s) (47.84%), neck (44.87%), back (35.54%) and shoulder (29.61%). In last year, workers reported that musculoskeletal disorders of lumbar (64.13%), knee(s) (41.68%), neck (33.27%) and back (31.06%) respectively caused limitation in their function. Details of other musculoskeletal disorders prevalence were reported in Table 2 and Table 3.

**Table 2:** Musculoskeletal disorders’ prevalence at recent week in our subjects with musculoskeletal disorders (n=666).

**Body region**	**Frequency**	**Percentage**
Lumbar	425	63.81
One or both knees	302	45.35
Neck	265	39.79
Upper back	200	30.03
Shoulder	189	28.38
Wrist/had	175	26.28
One or both ankle/feet	147	22.07
One or both hips/things	107	16.07
Elbow	99	14.87

**Table 3:** Musculoskeletal disorders prevalence at recent year in our subjects with musculoskeletal disorders (n=878).

**Body region**	**Frequency**	**Percentagr**
Lumbar	563	64.12
One or both knees	420	47.84
Neck	394	44.87
Upper back	312	35.53
Shoulder	260	29.61
Wrist/hand	226	25.74
One or both ankle/feet	190	21.64
One or both hips/thighs	158	17.99
Elbow	108	12.30

In correlation analysis between age and BMI of workers with musculoskeletal disorders, the prevalence of participant workers in last week and year, musculoskeletal disorders prevalence had significant association with BMI (p=0.004) and non-significant association with age (p=0.084) of works.

According to career duration, our participants were divided into three groups: Less than 5 years, 5-20 years and more than 20 years. Prevalence of musculoskeletal disorders of last week in participants with less than five and more than 20 years career duration were significantly higher than participants with 5-20 years career duration (p=0.003). A similar significant difference was seen in prevalence of musculoskeletal disorders of last year (p=0.00) (Table 4). In our logistic regression analysis after entering demographic data into the model, age and past job history remained in our model and in the other hand, other demographic factors did not have significant impact on musculoskeletal disorders (Table 5).

**Table 4:** Musculoskeletal disorders prevalence at recent one week and year in our subjects according their age, BMI and work duration.

	**Work duration**	**MSD (%)**		**P value^a^**
		**Positive**	**Negative**	
Work duration	< 5 years	58.6	41.4	0.003
Recent Week	5-10 years	54.7	45.3	
	10-15 years	58.9	41.1	
	15-20 years	56.8	43.2	
	>20 years	46.2	53.8	
Recent year	< 5 years	43.2	56.8	0.00
	5-10 years	42.9	57.1	
	10-15 years	43.3	56.7	
	15-20 years	43.7	56.3	
	>20 years	29.4	70.6	
Age groups	<25	63.2	36.8	0.19
Recent Week	25-34	54.8	45.2	
	35-44	54.3	45.7	
	45-59	48.8	51.2	
	>60	40	60	
Recent year	<25 25-34	47.1 41.7	52.9 58.3	0.21
	35-44	37.4	62.6	
	45-59	36	64	
	>60	20	80	
BMI groups Recent week	<20 20-24	49 59.3	51 40.7	0.00
	25-29	49.5	50.5	
	>30	52.3	47.7	
Recent year	<20 20-24	26.5 44.6	37.5 55.4	0.00
	25-29	35.8	64.2	
	>30	39.2	60.8	

^a^ all P values were calculated with Chi-Square test.

**Table 5:** Results of regression analysis in our participants.

****	**Beta**	**Standard Error**	**Significance**	**95.0% CI for EXP(B)**	
				**Upper**	**Lower**
Constant	0.19	0.16	0.24	-0.13	0.50
Age	-0.006	0.003	0.04	-0.01	0.00
Sex	0.10	0.07	0.19	-0.05	0.25
Dominant hand	0.01	0.05	0.76	-0.08	0.10
Past job history	0.01	0.03	0.001	0.004	0.02
Body mass index	0.004	0.004	0.24	-0.003	0.01

## Discussion

Findings of our study showed that 46.3% of workers in last week and 61% of workers in last year had claimed one of musculoskeletal disorders in their work places. Lumbar, knee(s) and neck had most common musculoskeletal disorders prevalence in the last week and year. Musculoskeletal disorders in last week and year had significant association with job duration and BMI in our workers. In our searching on the literature, Ford et al. in their study on 1566 iron workers in United States reported back rejoin (56%) as the highest musculoskeletal disorders’ prevalence.[21] Choi et al. reported that regardless of body part, the prevalence of musculoskeletal disorders was 25.5% among 2093 aging male steel workers.[22] In comparison with these studies, the prevalence of musculoskeletal disorders in our workers was higher than other studies. Substandard work places and inattention of workers to the caution instructions without national and effective preventive strategies or programs might be responsible for this higher rate. One of the other explanation of this difference comes back to sample size and selection method of workers. On the other hand, the epidemiological standards must be similar for better comparison. We did not find a similar study with our work and different inclusion criteria in noted study might have impact on reported musculoskeletal disorders’ prevalence.

Lumbar, knee(s) and back symptoms were found to be the most frequent problem among the workers studied. This high prevalence might be due to awkward working postures, manual material handling and long hours of standing work, which were common at almost all workstations and job activities observed. More complaints in lumbar and back were accompanied with the highest rates of sick leave. We suggested that next interventional programs for prevention of occupational injuries in workplaces of workers of steel companies must focus on reducing physical exposure to the musculoskeletal disorders risk factors of these regions.

Findings of the present study showed that job duration and BMI were significantly associated with musculoskeletal symptoms in the different body regions. BMI of workers had a role in improving their efficacy and obese workers had higher chance of musculoskeletal disorders and work related trauma. We need to ergonomic programs for change BMI of our workers and help us to achieve suitable BMI and physical fitness. Previous researches showed that recently employed workers in a company had more chance to encounter with occupational injuries than workers who have been employed for longer period of time.[23][24][25][26] Patients with lower job duration did not have enough experience for to meet risk factors because this situation had impacts on their interactions with workplaces and other workers and knowing about surrounding hazards.[23] Other potential explanation of reporting lower musculoskeletal disorders’ prevalence in workers with high job duration might be due to selection bias in our workers selecting method. On the other hand, workers who had musculoskeletal disorders did not remain in workplace and only healthy workers without musculoskeletal disorders participated in this study. Safety and decreased rate of musculoskeletal disorders in steel industry are related to interaction between workers and potential hazards of their environment.[27] We need careful evaluations of steel workers and their workplaces for gathering more information to support or reject this idea.

Among ergonomic risk factors such as awaked posture, repetitive motions, forceful excretion were present in our workstation in Iranian steel industries.

Our study had some limitations. First, most of our participants were male, second, due to self reporting nature of Nordic questionnaire, the educational level of the respondent may affect the completion of questionnaire and third, we did not have any measurement scale for measuring the intensity of the pain/discomfort which was reported by respondents.

It was concluded that musculoskeletal disorders in our steel company happened in high rate. We recommended additional studies to be performed for accurate assessment of musculoskeletal disorders risk factors. Noted programs must focus on reducing physical exposure to the musculoskeletal disorders risk factors of these regions.
